# Combination of echocardiography with systemic hemodynamic parameters for early risk stratification of hemodynamically significant patent ductus arteriosus in preterm infants

**DOI:** 10.3389/fped.2025.1616706

**Published:** 2025-09-23

**Authors:** Cuie Chen, Yuechong Cui, Shujun Chen, Jiaonv Chen, Lirong Zhao, Yuanyuan Sun, Liuqing Ji, Guoliang Wang

**Affiliations:** ^1^ Department of Neonatology, Yiwu Maternity and Children Hospital, Jinhua, Zhejiang, China; ^2^ Department of Child Healthcare, Yiwu Maternity and Children Hospital, Jinhua, Zhejiang, China; ^3^ Department of Pediatrics, The Quzhou Affiliated Hospital of Wenzhou Medical University Quzhou People’s Hospital, Quzhou, Zhejiang, China; ^4^ Department of Pediatrics, the First Affiliated Hospital of Wenzhou Medical University, Wenzhou, Zhejiang, China; ^5^ Department of Vascular Surgery, the First Hospital of Jiaxing, Affiliated Hospital of Jiaxing University, Jiaxing, China

**Keywords:** systemic hemodynamic parameters, hemodynamically significant patent ductus arteriosus, echocardiography, preterm infants, risk stratification

## Abstract

**Background:**

Hemodynamically significant patent ductus arteriosus (hsPDA) is a major contributor to morbidity and mortality in extremely preterm infants. Both echocardiographic assessment and systemic hemodynamic monitoring have emerged as valuable tools for evaluating cardiovascular status during the early postnatal period. This study aimed to evaluate whether echocardiographic and systemic hemodynamic parameters within 72 hours can predict the development of hsPDA in preterm infants.

**Methods:**

In this prospective study, 98 infants born at our institution between October 2022 and March 2025 were enrolled based on inclusion criteria of gestational age ≤32 weeks and birth weight ≤1,500 g. Hemodynamic monitoring was conducted using the Non-Invasive Cardiac System (NICaS) at 24, 48, and 72 hours after birth, each followed immediately by echocardiographic evaluation.

**Results:**

Among 98 preterm infants, 85 had patent ductus arteriosus (PDA) at 24 hours, with 30 progressing to hsPDA. The hsPDA group had significantly lower gestational age and birth weight. Maternal eclampsia or preeclampsia, placental abruption, neonatal asphyxia, alveolar surfactant need, mechanical ventilation within 72 hours, and higher fluid intake in the first 24 hours were more frequent in this group. These infants required prolonged respiratory support and parenteral nutrition, and showed higher rates of intraventricular hemorrhage (IVH) and bronchopulmonary dysplasia (BPD). Compared to non-hsPDA infants, those with hsPDA had larger ductus arteriosus (DA) diameters, higher DA diameter/weight ratios at 48 and 72 hours, and elevated left atrium-to-aortic root (LA/Ao) ratios at 24, 48, and 72 hours. Stroke index (SI), cardiac output index (CI), and total body water percent (TBW%) were increased, while total peripheral resistance index (TPRI) was reduced at 48 and 72 hours. Multivariate analysis identified maternal eclampsia/preeclampsia, surfactant use, DA diameter-to-weight ratio, LA/Ao, and TBW% at 48 and 72 hours as independent risk factors. A combined model achieved high predictive accuracy (AUC = 0.981, sensitivity = 100%, specificity = 90.0%).

**Conclusion:**

This study demonstrated that combining echocardiographic parameters with systemic hemodynamic indicators at 72 hours of life provides significant predictive value for identifying preterm infants with a gestational age ≤32 weeks and birth weight ≤1,500 g who are at risk of developing hsPDA.

## Introduction

The ductus arteriosus (DA), a fetal shunt bypassing the lungs, normally closes after birth: functional closure via oxygen-induced constriction occurs within hours, followed by permanent anatomical closure over weeks (1). In preterm infants, developmental immaturity and perinatal factors (stress, infection, ventilation, fluid overload) often prevent closure, causing patent ductus arteriosus (PDA) (2). A persistent left-to-right shunt can lead to hemodynamically significant PDA (hsPDA) (3). PDA prevalence inversely correlates with gestational age and birth weight, affecting ∼65% of very low birth weight infants (<1,500 g) (4). hsPDA prevalence reaches 50% in preterm infants with a gestational age <28 weeks (5). Persistent hsPDA is associated with substantial morbidity, including pulmonary hemorrhage, heart failure, intraventricular hemorrhage, bronchopulmonary dysplasia, necrotizing enterocolitis, and increased mortality (6–9). Primary interventions include fluid restriction, ventilation support, and non-steroidal anti-inflammatory drugs (NSAIDs), with surgical ligation reserved for failures (10). However, both pharmacological and surgical treatments carry potential complications (11, 12). These significant risks underscore the importance of preventing and early identifying hsPDA.

Excessive early postnatal fluid intake increases PDA persistence risk in preterm infants (13–15). Mirza H et al. (16) linked fluid overload in the first 48 hours to prolonged hsPDA in infants <29 weeks. Bell et al. (13) reported that fluid restriction reduces PDA, BPD, and IVH incidence in comprehensive systematic review. However, excessive restriction risks compromised organ perfusion and inadequate nutrition, necessitating individualized fluid management guided by evidence (17). Preterm infants with PDA exhibit higher baseline cardiac output (CO) than those without; ibuprofen non-responders show higher CO than responders (18). Significant reductions in cardiac output index occur post-ibuprofen treatment (19) and post-surgical ligation (20). Comprehensive evaluation of fluid status and systemic hemodynamics is therefore essential for assessing hsPDA risk and guiding therapy decisions.

While echocardiography assesses cardiac structure and DA size, it cannot fully evaluate fluid status or hemodynamics. NICaS, a non-invasive bioimpedance device, effectively monitors fluid balance and systemic hemodynamics in neonates (21, 22). Studies have shown that NICaS correlates strongly with transpulmonary thermodilution in evaluating cardiac function in patients, the latter being regarded as the gold-standard invasive method (23, 24). Several studies have demonstrated the feasibility, reproducibility, and moderate correlation of NICaS measurements with echocardiographic parameters in neonates, including preterm infants. For example, Beck R et al. (25) reported no significant differences in HR, SVI, or CI between NICaS and CardioQ during simultaneous pre-/intra-operative monitoring in pediatric surgical patients (neonates to children), with strong inter-device CI correlation (r = 0.85). Wu W et al. (26) found no significant differences in HR, RR, SV, or CO when performing hemodynamic measurements using both NICaS and echocardiography simultaneously in preterm and term neonates. Recent evidence indicates that hemodynamic parameters obtained with NICaS monitoring are associated with hsPDA. Rabin et al. (27) identified significant variations in hemodynamic parameters according to PDA diameter among preterm infants born before 35 weeks’ gestation. Specifically, as PDA diameter increased, both the stroke index (SI) and CI increased significantly, while the total peripheral resistance index (TPRI) decreased.

This study conducted a comprehensive evaluation of echocardiographic parameters and systemic hemodynamic parameters derived from the NICaS in preterm infants born at ≤32 weeks of gestation with birth weights ≤1,500 g. The aim was to perform risk stratification in preterm infants at risk of hsPDA.

## Methods

### Study design and participants

The study included 110 infants born at our hospital between October 2022 and March 2025 with a gestational age ≤32 weeks and a birth weight ≤1,500 g. Infants were excluded if they had chromosomal or genetic abnormalities, complex or ductus-dependent congenital heart disease, a large atrial septal defect (ASD) (≥5 mm) or ventricular septal defect (VSD) (≥5 mm), were classified as small for gestational age (SGA) or large for gestational age (LGA), had severe congenital anomalies of other organ systems, or died or were discharged within seven days of birth.

**Figure 1:**
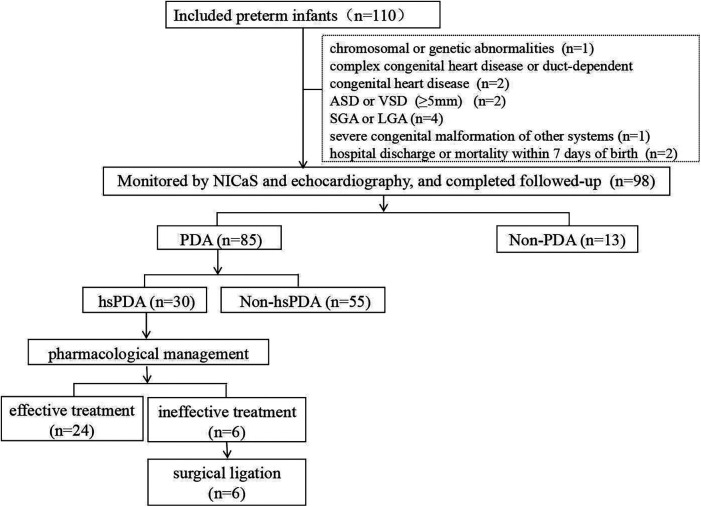
Flowchart of the study.

Finally, 12 preterm infants were excluded and 98 preterm infants were enrolled in the study. The flowchart of the study is presented in Figure 1. This prospective study was approved by the Ethics Committee of Yiwu Maternity and Children Hospital, China (No. A000152). Informed consent was obtained from the parents or legal guardians of all participating infants prior to enrollment.

### Study procedures

**Figure 2:**
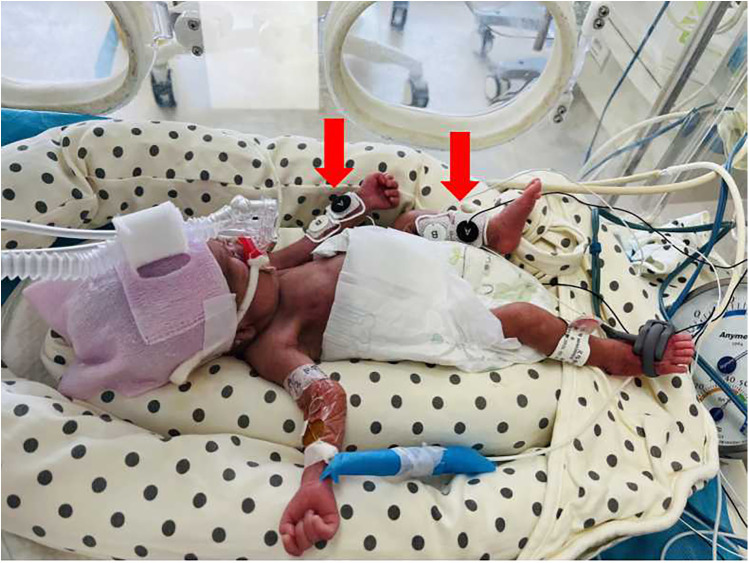
Electrode placement for NICaS assessment in a preterm infant. Electrode positions are indicated by red arrows.

Hemodynamic parameters in preterm infants were serially monitored using the NICaS (NI Medical, Petach Tikva, Israel) at critical postnatal time points: 24, 48, and 72 hours. All NICaS assessments were performed while the infants were in a quiet state. Electrodes were applied in a wrist-to-ankle configuration (Figure 2), specifically above the radial artery on the inner wrist and the posterior tibial artery on the inner ankle. Sensors were placed on two limbs, avoiding the simultaneous use of both lower limbs. If a limb had abnormal conditions, such as peripheral arterial disease, severe edema, or skin damage, an alternative limb was selected. NICaS measurements were recorded every 5 minutes, and the average of three consecutive readings was used for analysis. The parameters collected included Heart Rate (HR), Stroke Volume (SV), SI, CO, CI, Cardiac Power Index (CPI), Granov-Goor Index (GGI), Total Peripheral Resistance (TPR), TPRI, and Total Body Water (TBW), TBW as a Percentage of Body Weight (TBW%). Immediately following each NICaS assessment, echocardiography (CJ2-08000237, M9 T, Mindray, China) was performed. To minimize bias, all echocardiographic evaluations were conducted by a single experienced echocardiographer blinded to the NICaS data. Clinical data were collected from medical records by the research physicians.

hsPDA was defined between postnatal days 3 and 7 based on a combination of clinical signs and echocardiographic findings, in accordance with previously published and widely accepted criteria (3, 28, 29). Clinical signs included a characteristic systolic murmur, tachycardia, widened pulse pressure, systemic hypotension, and/or worsening respiratory status. Hypotension was defined as systolic blood pressure falling below the 5th percentile for gestational age and postnatal age, OR a ≥20% decrease from baseline systolic blood pressure requiring clinical intervention (e.g., fluid bolus or vasoactive medications). Worsening respiratory status was defined as an escalation in respiratory support [e.g., transition from nasal cannula to CPAP, CPAP to mechanical ventilation, or increasing mechanical ventilation parameters (FiO₂increase by ≥0.2 or mean airway pressure increase by ≥2 cmH_2_O)], OR new onset of respiratory acidosis attributable to hemodynamically significant PDA. Echocardiographic criteria consisted of a left-to-right shunt through the ductus arteriosus with an internal diameter ≥1.5 mm, along with at least one of the following: (1) left atrium-to-aortic root ratio (LA/Ao) ≥1.5; (2) diastolic flow velocity in the left pulmonary artery >0.2 m/s; (3) absent or reversed diastolic flow in the descending aorta. The primary outcome was the diagnosis of hsPDA based on these predefined criteria during the postnatal days 3–7.

For the pharmacological management of hsPDA, oral ibuprofen therapy is initiated after the careful exclusion of contraindications such as thrombocytopenia (platelet count <50 × 10⁹/L), impaired renal function (serum creatinine >141.5 μmol/L), necrotizing enterocolitis (NEC), and overt clinical bleeding, such IVH (grade III–IV), pneumorrhagia. Ibuprofen (Shanghai Johnson & Johnson Pharmaceuticals Ltd.; specification: 30 ml: 0.6 g) is administered via a nasogastric tube. First treatment course: an initial dose of 10 mg/kg is followed by two subsequent doses of 5 mg/kg each, administered every 24 hours. If there is an inadequate response, a second course is initiated: the initial dose is increased to 20 mg/kg, followed by two doses of 10 mg/kg each, also given at 24-hour intervals (11). When there is no response to the second course of ibuprofen, paracetamol at the dose of 15 mg/kg every 6 hours for 3 days can be tried (30). For hsPDA unresponsive to pharmacological treatment, surgical ligation is performed (31).

## Statistical analysis

Statistical analyses were performed using SPSS version 25.0 (SPSS Inc., Chicago, IL, USA). Continuous variables with a normal distribution were presented as the mean ± standard deviation (SD), while non-normally distributed data were expressed as the median and interquartile range [M (IQR)]. Categorical variables were expressed as frequencies (percentages). Continuous variables were analyzed using independent-sample *t*-tests or Mann–Whitney *U*-tests, as appropriate. Categorical variables were analyzed using the chi-square test, continuity-corrected chi-square test, or Fisher's exact test. Multivariate logistic regression analysis was performed to identify independent risk factors. A *P*-value < 0.05 was considered statistically significant. The predictive performance of the multivariable model was assessed using receiver operating characteristic (ROC) curve analysis and the area under the curve (AUC). Cutoff values were determined from the ROC curves of individual variables and outcomes, with the optimal threshold defined as the point corresponding to the maximum Youden Index, balancing sensitivity and specificity.

### Sample size and power analysis

Based on previous studies (32, 33), the expected incidence of hsPDA in infants ≤32 weeks and ≤1,500 g was set at 35%, and the anticipated AUC for the predictive model was assumed to be 0.9, compared with the null hypothesis value of 0.8. Using PASS 2021 software (NCSS, LLC, Kaysville, UT, USA) with a two-sided α = 0.05 and 80% power, the minimum required sample size was estimated to be 130 infants.

In the present study, a total of 98 preterm infants were enrolled. Among them, 30 had hsPDA and 55 did not. Based on a two-sided α of 0.05, with a null hypothesis AUC of 0.85 and an observed AUC of 0.981, the calculated statistical power was 0.98384. This indicated that the achieved statistical power met the predefined requirement.

Furthermore, the sample size in our study is comparable to those reported in previous literature (34, 35).

## Results

### Patients characteristics

Among the 98 preterm infants with a gestational age ≤32 weeks and birth weight ≤1,500 g included in the study cohort, 85 exhibited PDA at 24 hours postnatally. Of those with persistent PDA, 30 were definitively diagnosed with hsPDA at a median of 4 days (IQR 3-5.25 days) postnatally, followed by conservative management including fluid restriction, ventilator parameter adjustments, etc., with initial pharmacological intervention administered at a median of 6 days (IQR 5-8 days). Following a standardized course of ibuprofen therapy, successful ductal closure was achieved in 24 cases. The remaining 6 infants ultimately required surgical ligation to resolve the persistent shunt (Figure 1).

Baseline demographic and clinical characteristics of the preterm infants are presented in Table 1. Compared with the non-PDA group, the PDA group had a significantly lower gestational age (29.20 ± 1.74 weeks vs. 30.36 ± 0.91 weeks; *p* = 0.022). No statistically significant differences were observed between the groups in other clinical characteristics. A detailed comparative analysis of clinical parameters specific to hsPDA presentation is provided in Table 2. Preterm infants in the hsPDA group had significantly lower gestational age (28.43 ± 1.37 weeks vs. 29.64 ± 1.79 weeks; *p* = 0.002) and lower birth weight (1,138.67 ± 203.69 g vs. 1,232.22 ± 188.69 g; *p* = 0.037) compared to those in the non-hsPDA group. Infants with hsPDA also exhibited significantly higher rates of maternal eclampsia or preeclampsia (*p* = 0.019), placental abruption (*p* = 0.031), neonatal asphyxia (*p* = 0.007), alveolar surfactant requirement (*p* < 0.001), invasive mechanical ventilation within 72 hours postpartum (*p* = 0.001), and greater fluid intake during the first 24 hours after birth (*p* = 0.025) compared to the non-hsPDA group. Furthermore, preterm infants with hsPDA had significantly longer durations of mechanical ventilation (*p* < 0.001), extended oxygen therapy requirements (*p* < 0.001), and prolonged dependence on parenteral nutrition (*p* < 0.001), along with higher incidence rates of IVH (Grade II–IV) (*p* = 0.035) and BPD (*p* = 0.001) compared to their non-hsPDA counterparts.

**Table 1:** Baseline demographic and clinical characteristics of the preterm infants with or without PDA.

Clinical characteristics	PDA (*n* = 85)	Non-PDA (*n* = 13)	T, Z or *X*^2^	*P*
Male	40 (47.1%)	8 (61.5%)	0.946	0.331
Gestational age (weeks)	29.20 ± 1.74	30.36 ± 0.91	−3.657	0.001**
Birth weight (grams)	1,195.29 ± 199.21	1,176.15 ± 217.89	0.318	0.751
Maternal age (years)	31.27 ± 6.19	32.77 ± 5.42	−0.825	0.411
Eclampsia or preeclampsia	20 (23.5%)	4 (30.8%)	0.048^a^	0.827
Gestational diabetes mellitus	21 (24.7%)	6 (46.2%)	2.599	0.107
antenatal steroid administration	45 (52.9%)	5 (38.5%)	0.946	0.331
Placental abruption	13 (15.3)%	4 (30.8%)	1.419^a^	0.234
PROM (h)	0 (0, 24)	0 (0, 37.5)	−0.539	0.590
Neonatal asphyxia	22 (25.9%)	2 (15.4%)	0.410^a^	0.522
Alveolar surfactant requirement	45 (52.9%)	4 (30.8%)	1.419^a^	0.234
EOS	48 (56.5%)	5 (38.5%)	1.473	0.225
Invasive mechanical ventilation within 72 h after birth	24 (28.2%)	2 (15.4%)	0.410^a^	0.522
Mechanical ventilation (d)	14 (8, 26)	12 (7, 21)	−0.598	0.550
Oxygen therapy (d)	30 (17, 41)	27 (21, 39)	−0.037	0.971
Parenteral nutrition (d)	25 (17.5, 35.5)	27.5 (17.25, 35.25)	−0.199	0.842
IVH (Grade II-IV)	6 (7.5%)	0	–^b^	1.000
NEC	2 (2.4%)	2 (15.4%)	2.129^a^	0.145
ROP	9 (10.6%)	1 (7.7%)	0.000^a^	1.000
BPD	26 (30.6%)	4 (30.8%)	0.000^a^	1.000

PDA, patent ductus arteriosus; PROM, premature rupture of fetal membranes; EOS, early-onset sepsis; IVH, intraventricular hemorrhage; NEC, necrotizing enterocolitis; ROP, retinopathy of prematurity; BPD, bronchopulmonary dysplasia.

^a^ corrected *χ*^2.^

^b^ Fisher's exact probability method.

**P < 0.01.

**Table 2:** Baseline demographic and clinical characteristics of the preterm infants with or without hsPDA.

Clinical characteristics	hsPDA (*n* = 30)	Non-hsPDA (*n* = 55)	T, Z or *X*^2^	*P*
Male	15 (50%)	25 (45.5%)	0.161	0.688
Gestational age (weeks)	28.43 ± 1.37	29.64 ± 1.79	−3.204	0.002**
Birth weight (grams)	1,138.67 ± 203.69	1,232.22 ± 188.69	−2.116	0.037*
Maternal age (years)	30.00 ± 5.82	31.87 ± 6.35	−1.332	0.187
Eclampsia or preeclampsia	11 (36.7%)	8 (14.5%)	5.473	0.019*
Gestational diabetes mellitus	10 (33.3%)	11 (20.0%)	1.855	0.173
Antenatal steroid administration	18 (60%)	26 (47.3%)	0.946	0.331
Placental abruption	8 (26.7%)	5 (9.1%)	4.629	0.031*
PROM (h)	0 (0, 11.25)	0 (0, 24)	−0.582	0.561
Neonatal asphyxia	13 (43.3%)	9 (16.4%)	7.360	0.007**
Alveolar surfactant requirement	25 (83.3%)	20 (36.4%)	17.190	0.000**
EOS	17 (56.7%)	31 (56.4%)	0.001	0.979
Invasive mechanical ventilation within 72 h postpartum	15 (50%)	9 (16.4%)	10.839	0.001**
Mechanical ventilation (d)	26.5 (15.75, 38.75)	11.0 (7.0, 15.0)	−4.341	0.000**
Oxygen therapy (d)	40.5 (20.0, 57.25)	11.0 (7.0, 15.0)	−3.556	0.000**
Parenteral nutrition (d)	35.5 (24.75, 46.5)	21 (16.0, 29.0)	−3.608	0.000**
Fluid intake in 0–24 h (ml/kg)	85.40 ± 7.65	81.57 ± 7.19	2.283	0.025*
Fluid intake in 24–48 h (ml/kg)	92.83 ± 9.53	89.87 ± 7.50	1.573	0.120
Fluid intake in 48–72 h (ml/kg)	98.67 ± 12.38	98.15 ± 7.79	0.236	0.814
Urine volume in 0–24 h (ml/kg.h)	3.08 ± 1.25	2.65 ± 0.94	1.737	0.086
Urine volume in 24–48 h (ml/kg.h)	3.24 ± 1.31	2.97 ± 1.00	1.064	0.290
Urine volume in 48–72 h (ml/kg.h)	2.27 ± 0.92	2.68 ± 1.01	−1.855	0.067
Use of inotropes/vasopressors	8 (26.7%)	6 (10.9%)	3.503	0.061
pulmonary hemorrhage	1 (3.3%)	1 (1.8%)	0.000^a^	1.000
IVH (Grade II-IV)	5 (16.7%)	1 (1.8%)	4.457^a^	0.035*
NEC	1 (3.3%)	1 (1.8%)	0.000^a^	1.000
ROP	3 (10%)	6 (10.9%)	0.000^a^	1.000
BPD	16 (53.3%)	10 (18.2%)	11.297	0.001**

hsPDA, hemodynamically significant patent ductus arteriosus; PROM, premature rupture of fetal membranes; EOS, early-onset sepsis; IVH, intraventricular hemorrhage; NEC, necrotizing enterocolitis; ROP, retinopathy of prematurity; BPD, bronchopulmonary dysplasia.

^a^ corrected *χ*^2^.

* *p* < 0.05.

** *p* < 0.01.

### Comparative analysis of echocardiographic and hemodynamic parameters in preterm infants with vs. without hsPDA

Infants in the hsPDA group demonstrated significantly larger DA diameters and higher DA diameter-to-weight ratios at both 48 and 72 hours postnatally compared to those in the non-hsPDA group (all *p* < 0.05), as detailed in Table 3. Notably, the LA/Ao ratio was also significantly elevated in the hsPDA cohort at both 48 and 72 hours compared with non-hsPDA controls (all *p* < 0.05). In addition, significant hemodynamic differences were observed between the two study groups. Specifically, preterm infants in the hsPDA group exhibited elevated SI and CI, along with reduced TPRI and increased TBW% at both 48 and 72 hours postnatally compared to the non-hsPDA group (all *p* < 0.05). We performed three separate multivariate regression analyses based on clinical variables and hemodynamic screening time points. Due to collinearity between gestational age and birth weight, only gestational age was included in the regression models. Multivariate logistic regression revealed that eclampsia or preeclampsia, alveolar surfactant requirement, DA diameter-to-weight ratio, LA/Ao ratio, and TBW% at 48 and 72 hours were independent risk factors for hsPDA (Tables 4–6).

**Table 3:** Echocardiographic and hemodynamic parameters in preterm infants with and without hsPDA at24, 48 and 72 hours postnatally.

Parameters	hsPDA (*n* = 30)	Non-hsPDA (*n* = 55)	*X^2^or T*	*P*
at 24h				
DA diameter (mm)	2.35 ± 0.83	2.40 ± 0.68	−0.286	0.775
DA diameter/weight(mm/kg)	1.97 ± 0.92	1.83 ± 0.85	0.680	0.498
LA/Ao	1.35 ± 0.23	1.23 ± 0.17	2.429	0.018*
SV (ml)	3.22 ± 2.80	3.03 ± 0.88	0.466	0.642
SI (ml/m^2^)	26.12 ± 6.65	25.62 ± 6.58	0.328	0.744
CO (L/min)	0.45 ± 0.39	0.40 ± 0.12	0.873	0.385
CI (L/min/m^2^)	3.66 ± 1.01	3.39 ± 0.99	1.168	0.246
CPI (w/m^2^)	0.32 ± 0.10	0.32 ± 0.10	0.109	0.913
GGI	12.03 ± 2.35	13.16 ± 2.95	−1.816	0.073
TPR (dn*s/cm^5^)	8,651.72 ± 3,339.29	9,100.57 ± 2,729.47	−0.669	0.505
TPRI (dn*s/cm^5^*m^2^)	932.86 ± 358.05	1,072.75 ± 348.99	−1.750	0.084
TBW (kg)	1.14 ± 1.02	1.08 ± 0.12	0.327	0.746
TBW %	86.57 ± 5.75	85.28 ± 2.07	1.502	0.137
At 48h				
DA diameter (mm)	2.54 ± 0.70	2.10 ± 0.84	2.420	0.018*
DA diameter/weight(mm/kg)	2.18 ± 0.72	1.75 ± 0.74	2.561	0.012*
LA/Ao	1.34 ± 0.26	1.21 ± 0.24	2.268	0.026*
SV (ml)	2.78 ± 0.89	2.50 ± 0.79	1.517	0.133
SI (ml/m^2^)	26.27 ± 7.41	21.56 ± 6.14	3.138	0.002**
CO (L/min)	0.42 ± 0.14	0.36 ± 0.12	1.976	0.054
CI (L/min/m^2^)	3.98 ± 1.19	3.13 ± 0.91	3.676	0.000**
CPI (w/m^2^)	0.34 ± 0.11	0.31 ± 0.10	1.411	0.162
GGI	11.09 ± 2.64	10.46 ± 3.18	0.922	0.359
TPR (dn*s/cm^5^)	8,411.16 ± 3,520.53	11,857.18 ± 6,862.03	−3.059 8	0.003**
TPRI (dn*s/cm^5^*m^2^)	882.07 ± 375.13	1,325.53 ± 662.34	−3.940 8	0.000**
TBW (kg)	0.93 ± 0.11	1.04 ± 0.11	−4.661	0.000**
TBW %	88.04 ± 2.11	85.90 ± 1.88	4.802	0.000**
At 72h				
DA diameter (mm)	2.42 ± 0.84	1.29 ± 0.78	5.937	0.000**
DA diameter/weight(mm/kg)	2.06 ± 0.78	0.99 ± 0.78	6.041	0.000**
LA/Ao	1.41 ± 0.27	1.23 ± 0.32	2.380	0.021*
SV (ml)	2.96 ± 1.12	2.46 ± 0.53	2.264	0.030*
SI (ml/m^2^)	27.78 ± 8.07	23.09 ± 5.47	3.183	0.002**
CO (L/min)	0.45 ± 0.16	0.38 ± 0.10	2.041	0.048*
CI (L/min/m^2^)	4.20 ± 1.30	3.29 ± 0.81	3.472	0.001**
CPI (w/m^2^)	0.38 ± 0.16	0.32 ± 0.08	1.913	0.063
GGI	12.27 ± 4.10	11.18 ± 3.51	1.292	0.200
TPR (dn*s/cm^5^)	8,125.02 ± 3,067.22	10,059.82 ± 3,127.17	−2.74	0.007**
TPRI (dn*s/cm^5^*m^2^)	847.18 ± 317.02	1,147.42 ± 327.87	−4.08	0.000**
TBW (kg)	0.94 ± 0.14	1.07 ± 0.12	−3.42	0.001**
TBW %	90.28 ± 2.90	85.06 ± 2.35	7.506	0.000**

hsPDA, hemodynamically significant patent ductus arteriosus; DA, ductus arteriosu; LA/Ao, left atrium-to-aortic root; SV, stroke volume; SI, stroke index; CO, cardiac output; CI, cardiac output index; CPI, cardiac power index; GGI, Granov-Goor index; TPR, total peripheral resistance; TPRI, total peripheral resistance index; TBW, total body water; TBW%, total body water as a percentage of body weight.

* *P* < 0.05.

** *P* < 0.01.

**Table 4:** Multivariate logistic regression analysis of clinical risk factors associated with hsPDA.

Variables	B	SE	Wald	*p*	OR	95% CI
Gestational age (weeks)	−0.098	0.194	0.258	0.611	0.906	0.620–1.324
Eclampsia or preeclampsia	−1.368	0.678	4.064	0.044*	0.255	0.067–0.963
Placental abruption	−1.417	0.731	3.758	0.053	0.242	0.058–1.016
neonatal asphyxia	0.022	0.733	0.001	0.976	1.022	0.243–4.304
alveolar surfactant requirement	−1.838	0.725	6.418	0.011*	0.159	0.038–0.660
Invasive mechanical ventilation within 72 h after birth	−0.665	0.700	0.903	0.342	0.514	0.130–2.028
Fluid intake in 0–24 h (ml/kg)	0.020	0.041	0.231	0.631	1.020	0.942–1.104

SE, standard error; OR, odds ratio; 95% CI, 95% confidence interval; hsPDA, hemodynamically significant patent ductus arteriosus.

* *p* < 0.05.

**Table 5:** Multivariate logistic regression analysis of echocardiographic and hemodynamic predictors of hsPDA at 48 hours postnatally.

Variables	B	SE	Wald	*p*	OR	95% CI
DA diameter/weight(mm/kg)	1.049	0.434	5.835	0.016*	2.854	1.219–6.685
LA/Ao	2.426	1.190	4.159	0.041*	11.318	1.099–116.533
CI (L/min/m^2^)	0.343	0.456	0.567	0.451	1.409	0.577–3.441
TPRI (dn*s/cm^5^*m^2^)	−0.001	0.001	1.260	0.262	0.999	0.997–1.001
TBW %	0.536	0.153	12.264	0.000**	1.709	1.266–2.306

SE, standard error; OR, odds ratio; 95% CI, 95% confidence interval; hsPDA, hemodynamically significant patent ductus arteriosus; DA, ductus arteriosu; LA/Ao, left atrium-to-aortic root; CI, cardiac output index; TPRI, total peripheral resistance index; TBW%, total body water as a percentage of body weight.

* *p* < 0.05.

**Table 6:** Multivariate logistic regression analysis of echocardiographic and hemodynamic predictors of hsPDA at 72 hours postnatally.

Variables	B	SE	Wald	*p*	OR	95% CI
DA diameter/weight(mm/kg)	1.542	0.440	12.259	0.000**	4.673	1.971–11.079
LA/Ao	2.925	1.420	4.240	0.039**	18.626	1.151–301.315
CI (L/min/m^2^)	−0.261	0.676	0.149	0.699	0.770	0.205–2.897
TPRI (dn*s/cm^5^*m^2^)	−0.003	0.002	1.939	0.164	0.997	0.993–1.001
TBW %	0.534	0.142	14.688	0.000**	1.721	1.304–2.272

SE, standard error; OR, odds ratio; 95% CI, 95% confidence interval; hsPDA, hemodynamically significant patent ductus arteriosus; DA, ductus arteriosu; LA/Ao, left atrium-to-aortic root; CI, cardiac output index; TPRI, total peripheral resistance index; TBW%, total body water as a percentage of body weight.

**P < 0.01.

### Potential role in early prediction of hsPDA by combining echocardiographic and systemic hemodynamic parameters

**Figure 3:**
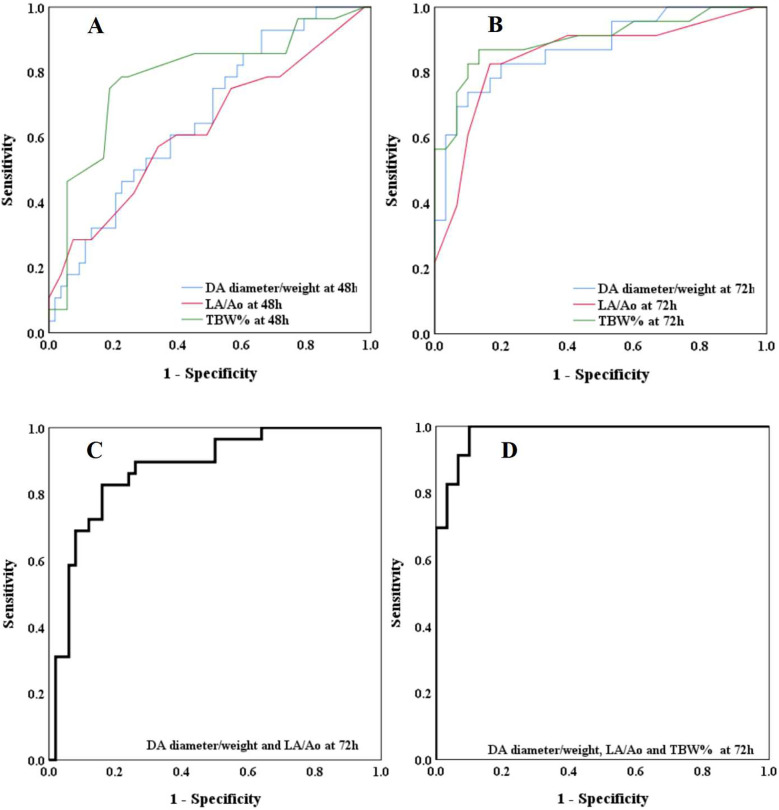
Receiver operating characteristic (ROC) curves for predicting hsPDA. **(A)** Predictive performance of DA diameter/weight ratio, LA/Ao ratio, and TBW% at 48 hours postnatal age. **(B)** Predictive performance of DA diameter/weight ratio, LA/Ao ratio, and TBW% at 72 hours postnatal age. **(C)** Combined model of DA diameter/weight ratio and LA/Ao ratio at 72 hours. **(D)** Combined model of DA diameter/weight ratio, LA/Ao ratio, and TBW% at 72 hours.

The ROC curves evaluating the predictive performance of ultrasound parameters (DA diameter/weight ratio and LA/Ao ratio) and hemodynamic parameters (TBW%) at 48 and 72 hours postnatal age for hsPDA are shown in Figure 3 and Table 7. Compared to measurements at 48 hours, echocardiographic and systemic hemodynamic parameters assessed at 72 hours demonstrated superior predictive ability for hsPDA, with larger AUCs, higher sensitivity, and greater specificity. At 72 hours, the DA diameter/weight ratio showed predictive value for hsPDA, with an AUC of 0.875, sensitivity of 73.9%, and specificity of 90.0% at a threshold of 1.64. The LA/Ao ratio also demonstrated significant predictive value, with an AUC of 0.850; at an optimal threshold of 1.29, it achieved 82.6% sensitivity and 83.3% specificity. TBW% showed strong predictive ability, yielding an AUC of 0.901. Using a cutoff of 87.01, sensitivity and specificity were 87.0% and 86.7%, respectively. Combining the DA diameter/weight ratio and LA/Ao ratio improved predictive accuracy, achieving an AUC of 0.874, with 82.8% sensitivity and 84.0% specificity. Incorporating all three parameters (DA diameter/weight ratio, LA/Ao ratio, and TBW%) further enhanced model performance, yielding an AUC of 0.981, with 100% sensitivity and 90.0% specificity at optimal thresholds of 1.64 (DA diameter/weight), 1.29 (LA/Ao), and 87.01 (TBW%).

**Table 7:** Predictive performance of individual and combined parameters (DA diameter/weight, LA/Ao ratio, TBW%) for hsPDA at 72 hours postnatally.

Parameters	AUC	Sensitivity (%)	Specificity (%)	Cut off point	*P*
at 48h					
DA diameter/weight (mm/kg)	0.664	92.9	34.0	1.53	0.016*
LA/Ao	0.628	57.1	66.0	1.33	0.059
TBW %	0.785	75.0	81.1	87.69	0.000**
At 72h					
DA diameter/weight (mm/kg)	0.875	73.9	90.0	1.64	0.000**
LA/Ao	0.850	82.6	83.3	1.29	0.000**
TBW %	0.901	87.0	86.7	87.01	0.000**
DA diameter/weight and LA/Ao	0.874	82.8	84.0	1.64 and 1.29	0.000**
DA diameter/weight, LA/Ao and TBW %	0.981	100	90.0	1.64, 1.29 and 87.01	0.000**

hsPDA, hemodynamically significant patent ductus arteriosus; DA, ductus arteriosu; AUC, area under the curve; LA/Ao, left atrium-to-aortic root; TBW%, total body water as a percentage of body weight.

* *P* < 0.05.

** *p* < 0.01.

## Discussion

Previous studies have demonstrated that hsPDA is associated with numerous adverse outcomes in preterm infants (7–9), and its effective prevention and management remain critical priorities for neonatologists. However, in the first few days of life, PDA may remain clinically “silent,” while its hemodynamic characteristics continue to evolve (36). To address this dynamic clinical challenge, we developed a novel predictive model that integrates both echocardiographic indices (such as DA diameter/weight ratio and LA/Ao ratio) and hemodynamic parameters (including TBW%). This composite model demonstrated high predictive accuracy, achieving 100% sensitivity and 94.4% specificity in identifying preterm infants with a gestational age ≤32 weeks and birth weight ≤1,500 g who are at high risk for hsPDA.

The observed median postnatal ages for definitive hsPDA diagnosis (4 days; IQR 3–5.25 days) and initial pharmacological intervention (6 days; IQR 5–8 days) highlight a critical window for early identification. These timings align with established literature (3, 28). Our model's exceptional performance (AUC 0.981) within the first 72 hours of life provides actionable risk stratification significantly before the median clinical diagnosis. This enables targeted monitoring, facilitating earlier confirmation in high-risk infants. Furthermore, accurate prediction during this pre-diagnostic phase and into the subsequent observation period can inform clinical decisions regarding conservative management duration vs. pharmacotherapy timing. A persistently high model-derived risk score may support earlier intervention in equivocal cases, while a low score could reinforce confidence in continuing conservative measures. Thus, integrating these clinical timelines underscores our model's effectiveness as a tool for enhancing early risk assessment and optimizing hsPDA management pathways within the established clinical course.

Studies have confirmed that DA diameter and DA diameter/weight were risk factors for developing hsPDA (27). The transductal shunt volume is primarily influenced by the luminal diameter of the DA (37). In the present study, we selected the DA diameter/weight ratio as the variable parameter rather than DA diameter alone, as this helps eliminate individual variability caused by differences in body weight. Storme et al. (38) demonstrated that a DA diameter/weight >1.4 mm/kg measured within the first 48 hours after birth serves as a reliable indicator for hsPDA diagnosis. Liu et al. (39) reported that a higher threshold of DA diameter/weight >3.2 mm/kg at 72 hours of postnatal age has predictive value for treatment-requiring PDA in very preterm infants (≤30 weeks’ gestation). Our study found that the DA diameter/weight >1.64 mm/kg at 72 hours could serve as a predictive factor for hsPDA in infants with a gestational age ≤32 weeks and birth weight ≤1,500 g. Meanwhile, we observed that the LA/Ao ratio was an independent risk factor for hsPDA. This finding is consistent with pathophysiological principles, as left atrial enlargement reflects volume overload secondary to left-to-right shunting through the DA, particularly in the absence of a significant intra-atrial shunt during the cardiovascular transition period (40). We found that an LA/Ao ratio >1.29 at 72 hours could serve as a risk factor for predicting hsPDA. While no formal guidelines exist for hsPDA diagnosis, treatment, or prevention, neonatologist-recognized research and consensus inform practice. These studies typically required treatment for preterm infants with hsPDA at LA/Ao ratios ≥1.4 or ≥1.5 (3, 30), thresholds higher than those identified in our study. This difference likely reflects our focus on risk-stratifying high-risk preterm infants for hsPDA rather than directly guiding pharmacological intervention. Furthermore, gestational age and birth weight variations may impact optimal cutoff values in the prediction model.

Our data demonstrated that infants who developed hsPDA exhibited distinct hemodynamic patterns compared to controls: TPRI, while SI, CI and TBW% increased at 48 hours. At 72 hours, SI, CI, and TBW% showed further elevation, accompanied by a sustained reduction in TPRI. These findings reflect the characteristic hemodynamic changes associated with PDA. When a PDA persists, a portion of the cardiac output is shunted through the ductus into the pulmonary circulation, thereby reducing effective systemic perfusion, particularly to the lower extremities. To compensate for this “ductal steal” phenomenon, the cardiovascular system initiates adaptive mechanisms: enhanced myocardial contractility and decreased peripheral vascular resistance lead to an increased SV and elevated CO, resulting in higher SI and CI. These changes help maintain adequate tissue perfusion despite the left-to-right shunting. The TBW% serves as an indicator of total body fluid content in preterm infants. Current clinical guidelines primarily advocate fluid restriction as a conservative therapeutic approach for managing hsPDA (17). This strategy is theorized to alleviate left atrial volume overload secondary to ductal shunting by reducing the burden on pulmonary circulation (6). However, this practice carries inherent risks, including potential compromise of adequate nutritional intake and impaired systemic circulatory function. In clinical practice, fluid administration in preterm infants typically begins at 60–80 ml/kg/day, with daily increments of 10–20 ml/kg/day to reach a target of approximately 150 ml/kg/day by postnatal days 5–7 (17, 41). For neonates with confirmed or evolving hsPDA, current evidence supports moderate fluid intake (120–130 ml/kg/day) to mitigate pulmonary congestion due to left heart volume overload (17), while maintaining cardiopulmonary balance. Despite strict fluid restriction protocols implemented at our center, a significant difference in TBW% was observed between the hsPDA and non-hsPDA groups. Preterm infants with higher TBW% demonstrated an increased susceptibility to developing hsPDA. The underlying pathophysiological mechanism may involve reduced systemic circulation secondary to PDA, accompanied by decreased urine output. Although the difference in urine volume between 48 and 72 hours postnatally was not statistically significant, a trend toward reduced urine output was noted in the hsPDA group compared to non-hsPDA counterparts. These findings suggest that elevated TBW% represents a clinically relevant marker associated with hsPDA development. Furthermore, our results demonstrate that combining the three parameters—DA diameter/weight, LA/Ao, and TBW%—at 72 hours postnatally significantly improves the predictive performance of our model. This temporal association aligns with the critical transitional window of cardiopulmonary adaptation, during which pulmonary vascular resistance declines sharply, coinciding with the terminal phase of ductal remodeling. These pathophysiological insights support the establishment of a targeted hemodynamic surveillance protocol during the 72-hour postnatal window. Early identification of these high-risk infants enables the implementation of stratified management strategies, allowing for targeted interventions such as fluid restriction in high-risk populations, while avoiding unnecessary therapies in those with self-limiting ductal shunts.

Although NICaS exhibits strong correlation with transpulmonary thermodilution in adults, its derived hemodynamic parameters are not interchangeable with those obtained via continuous thermodilution (24). Meanwhile, several factors have been identified that can influence the accuracy of hemodynamic measurements obtained using NICaS (23, 42, 43). Since NICaS measurements are sensitive to movement, patients should remain still during the procedure. In cases of severe tachycardia or arrhythmias, the bioimpedance method may produce inaccurate cardiac output estimates due to reduced blood flow during systole (42). In this study, all patients demonstrated normal heart rates during measurement. In cases of significant edema, baseline body impedance can be significantly reduced, compromising the accuracy of measurements (42). Fluid shifts including massive fluid loss, transfusion, or third-space losses can cause baseline impedance drift, introducing unaccounted measurement errors (43). Established exclusion criteria for NICaS use include: severe aortic stenosis, where CO is typically underestimated, and significant aortic regurgitation, where CO tends to be overestimated (23). Consequently, understanding these limitations and excluding unsuitable patients is essential for accurate NICaS assessment. Additional technical constraints exist, particularly in extremely low birth weight infants, where small body surface area, skin impedance, motion artifacts, and unstable peripheral vasomotor tone may affect monitoring accuracy, which was an area requiring further investigation.

Furthermore, the NICaS device estimates SV and CO using whole-body bioimpedance by detecting cyclic changes in body impedance associated with systolic ejection. In the presence of a systemic-to-pulmonary shunt (for example, a left-to-right patent ductus arteriosus in preterm infants), a portion of the left ventricular output is diverted into the pulmonary circulation. This redistribution makes NICaS-derived absolute CO values less representative of effective systemic flow and therefore may introduce bias in the absolute measurement. In this study, the NICaS results were interpreted alongside echocardiographic assessment, and we emphasized relative (such as between-groups) changes rather than isolated absolute CO values. Van Wyk L et al. (44) conducted a systematic review evaluating the accuracy and trending ability of electrical biosensing technology (EBT) for non-invasive cardiac output monitoring in neonates. The authors concluded that EBT demonstrated reasonable accuracy but poor precision and was not interchangeable with transthoracic echocardiography. However, there is currently no study assessing the heterogeneity of NICaS compared with standard methods such as transpulmonary thermodilution and transthoracic echocardiography in the presence of a left-to-right shunt due to PDA, and further validation against reference methods such as transpulmonary thermodilution across different shunt severities is desirable.

Several limitations should be acknowledged. First, the lack of an internationally accepted definition of hsPDA constrains the generalizability and comparability of clinical research until standardized diagnostic criteria are established. Second, although our predictive model incorporated hemodynamic parameters and echocardiographic indices, it did not include certain potentially relevant clinical characteristics. Future research should consider more granular stratification by gestational age and birth weight. Third, the single-center design and relatively small sample size may limit the robustness and external validity of our findings. Multicenter collaborations and/or extended study periods are warranted to obtain larger, more representative cohorts.

## Conclusion

This study demonstrated that combining echocardiographic parameters with systemic hemodynamic indicators at 72 hours of life provides significant predictive value for identifying preterm infants with a gestational age ≤32 weeks and birth weight ≤1,500 g who are at risk of developing hsPDA. This model provides a basis for fluid management in preterm infants, with the aim of reducing the occurrence of hsPDA.

## Data Availability

The original contributions presented in the study are included in the article/Supplementary Material, further inquiries can be directed to the corresponding authors.
